# Medical Advertisements

**Published:** 1878-04

**Authors:** 


### MEDICAL ADVERTISEMENTS.

Quacks and pretenders of all sorts and kinds advertise themselves into notoriety through the public press, gaining the confidence and patronage of the dear people, and then swindling them outrageously and unmercifully.

While this is being accomplished, the regularly educated man looks on, turns up his nose at the quack and his patron alike, and says, audibly, that advertising is wrong—it disgraces the profession, while *mentally*, he knows better and fairly aches to try the device of the quack.

The regular medical profession who belong to some medical society, are not permitted, (if they obey the code by which they consent to be governed), to advertise in any manner. The result is, the field is left open to the ignorant pretender to medical knowledge, who reaps an abundant harvest by reason of his monopoly in advertising his business. Many regular physicians believe that the only way to rout the quack is to fight him with his own weapons, using the daily papers in modestly advertising their specialties, and so directing people suffering from such ailments as the specialist treats, to competent aid. But the code of ethics forbids it, hence many respectable physicians seek to dodge this code, and advertise themselves indirectly, by publishing the proceedings of their society meetings in the daily papers, in which the titles of the papers read are given, and the wonderful successs that always attends the treatment recommended is carefully set forth; often enumerating the number of cures so obtained, and the authors' names printed in full. Other medical societies issue pamphlets of their proceedings, and many physicians have essays printed in pamphlet form, which, somehow or other, find their way into a great many households and so advertise the author. These pamphlets usually treat of some special ailment and set forth the success that the author has met with in its treatment, and, strange to say, if there is a patient possessed of the particular disease treated of, anywhere in the vicinity, he surely finds that pamphlet and is led accordingly to its author for treatment.

A society of physicians in Albany, recognizing the advantage of advertising in this manner, have issued a pamphlet, ostensibly to the profession, but as a large number are found in possession of the laity, afflicted ones at that, it is not reasonable to suppose that this circumstance is accidental. The pamphlet contains numerous laudatory letters of the ability and skill of the surgeon (who has opened a dispensary for the cure of special ailments), from eminent medical gentlemen throughout the State, the reading of which could not fail to convince a patient that he would fall into skillful and able hands should he patronize the institution mentioned.

Now, mind you, we do not say such advertising is wrong. By no means. We believe it to be right, and applaud and recommend the means. But what we *do* object to is that it should be accomplished in this *indirect* manner, *so as to avoid violating the code.* Or, if medical gentlemen choose to do their advertising in this manner, they should not condemn regularly educated and competent practitioners who prefer to do their advertising openly and legitimately. We hold that, for a competent medical specialist to advertise his specialty in the public prints, so calling the attention of the afflicted to his facilities for treating their cases, is right and entirely modest; provided it is done in an unostentatious manner, void of puffs, certificates of cure, and the usual methods of quacks and imposters. And we further believe that only by such advertising can the flow of patients be diverted from the charlatan to the educated and skillful medical man, and the sooner our profession ascertain this truth, the earlier will we rid ourselves of the multiplicity of quacks that infest almost every community.

